# Dominant-negative ATF5 rapidly depletes survivin in tumor cells

**DOI:** 10.1038/s41419-019-1872-y

**Published:** 2019-09-24

**Authors:** Xiaotian Sun, James M. Angelastro, David Merino, Qing Zhou, Markus D. Siegelin, Lloyd A. Greene

**Affiliations:** 1 Department of Pathology and Cell Biology, Columbia University, New York, NY 10032 USA; 2 Department of Molecular Biosciences, University of California, Davis School of Veterinary Medicine, Davis, CA 95616 USA; 3 Present Address: CMI Strategies, Boulogne-Billancourt, 80 rue Gallieni, cedex, France

**Keywords:** Drug development, Apoptosis

## Abstract

Survivin (BIRC5, product of the *BIRC5* gene) is highly expressed in many tumor types and has been widely identified as a potential target for cancer therapy. However, effective anti-survivin drugs remain to be developed. Here we report that both vector-delivered and cell-penetrating dominant-negative (dn) forms of the transcription factor ATF5 that promote selective death of cancer cells in vitro and in vivo cause survivin depletion in tumor cell lines of varying origins. dn-ATF5 decreases levels of both survivin mRNA and protein. The depletion of survivin protein appears to be driven at least in part by enhanced proteasomal turnover and depletion of the deubiquitinase USP9X. Survivin loss is rapid and precedes the onset of cell death triggered by dn-ATF5. Although survivin downregulation is sufficient to drive tumor cell death, survivin over-expression does not rescue cancer cells from dn-ATF5-promoted apoptosis. This indicates that dn-ATF5 kills malignant cells by multiple mechanisms that include, but are not limited to, survivin depletion. Cell-penetrating forms of dn-ATF5 are currently being developed for potential therapeutic use and the present findings suggest that they may pose an advantage over treatments that target only survivin.

## Introduction

The transcription factor ATF5 is over-expressed in a variety of cancer types and such over-expression correlates with poor prognosis and treatment resistance^1–18^. ATF5 is a member of the activating transcription factor family and possesses a basic leucine zipper domain that is required for association with selected partners as well as DNA binding and transcriptional activity^2–4,19–22^. To interfere with ATF5 function, we developed a dominant-negative (dn) form of this protein that retains the native leucine zipper, but is N-terminally truncated and modified in the basic domain to abolish DNA binding and to potentially extend the leucine zipper^23^. When expressed in tumor cells in vitro or in vivo, dn-ATF5 promotes their apoptotic death and causes tumor regression^2–4,9,10,13,24^. To generate a dn-ATF5 with therapeutic potential, we designed a form fused to a cell-penetrating “penetratin”^25^ domain that can be produced either as a recombinant protein^26^ or by synthesis^27^. The presence of the cell-penetrating domain permits the penetratin-dn-ATF5 fusion peptide (CP-dn-ATF5) to rapidly pass through tissue barriers and to enter cells^26^. Both recombinant and synthetic forms of CP-dn-ATF5 effectively promote apoptosis of a wide variety of tumor cells in vitro and in vivo^26,27^. In contrast, neither plasmid nor penetratin delivered dn-ATF5 appears to affect survival of non-transformed cells and there have been no evident side-effects in mice treated with either the recombinant or synthetic forms of CP-dn-ATF5 at doses that suppress tumor growth^2,24,26,27^.

The mechanisms by which dn-ATF5 promotes tumor cell death are only partially understood. dn-ATF5 decreases levels of anti-apoptotic proteins BCL2 and MCL1 in tumor cells by what appears to be both decreased transcription and elevated protein destabilization^5,27,28^. A preliminary survey of additional proteins that regulate cell survival and death indicated that dn-ATF5 may also affect expression of the inhibitor-of-apoptosis protein (IAP) family member survivin (BIRC5, product of the *BIRC5* gene). Like ATF5, survivin is highly expressed in multiple tumor types with little expression in most non-transformed cells^29^. High survivin expression in tumors is correlated with metastasis, resistance to treatment and poor prognosis^30,31^. In addition to its action as an inhibitor of apoptosis, biological roles for survivin that also appear to contribute to its actions in tumors include regulation of cell cycle and promotion of mitochondrial function^31^. Agents that directly or indirectly down-regulate survivin levels interfere with the proliferation of cancer cells and promote their apoptotic death and thus, given survivin’s absence from most non-transformed cells, it has been widely considered as an attractive potential target for cancer treatment^30–36^. Consequently, there has been substantial effort to identify/generate agents that suppress survivin expression in neoplasias^31,33–36^. To date, no such drug has reached clinical use beyond trials, neither as a mono- or combination therapy. Thus there is a continued need to identify agents that affect survivin expression and that have the potential to be used as safe cancer therapeutics.

## Materials and methods

### Cells culture and transfection

GBM12 cells were kindly supplied by Dr. Jann Sarkaria (Mayo Clinic). All other cell lines were obtained from the ATCC and authenticated by the supplier. All lines were grown in DMEM supplemented with 10% fetal bovine serum, 100 U/ml penicillin and 100 U/ml streptomycin. siUSP9X (#6308 S, Cell Signaling Technology, Danvers MA), siSurvivin (#6351, Cell Signaling Technology; (#4390824, Silencer Select S1458, Ambion), siRNA CTR (#6568, Cell Signaling Technology; Silencer^TM^ Select Negative Control, #4390843, Ambion) were transfected into cells using Oligofectamine™ Transfection Reagent (Invitrogen, Waltham MA) following the supplier’s protocols. All plasmids were transfected by using Lipofectamine™ 3000 (Invitrogen) following the supplier’s protocols.

### Plasmids

FLAG-tagged human survivin cDNA cloned into a pCMV6-entry vector was obtained from Origene, Rockville MD (#RC205935). The plasmid used for FLAG-survivin over-expression was pLVX-EF1α-IRES-mCherry (#631987, Takara Bio USA, Mountainview CA), a bicistronic lentiviviral vector allowing the expression of the transgene and mCherry under the control of the EF1-α promoter. FLAG-survivin was generated and cloned in the pLVX vector using primers AAGAATTC (EcoRI)ATGGGTGCCCCGACGTTG and AATCTAGA(XbaI)TTACTTATCGTCGTCATC. GFP-BCL2^37^ was a gift from Clark Distelhorst (Addgene plasmid # 17999; http://n2t.net/addgene:17999; RRID:Addgene_17999).

Indicated experiments employed wild-type and mutant pCMV-1A-3xFLAG-dn-ATF5. To generate these constructs, DNA optimized for human codon usage with a 5’- BamHI site and a 3’-XhoI site were synthesized as gBlock fragments (Integrated DNA Technologies Inc, Skokie IL) encoding the wild-type dn-ATF5 sequence, MASMTGGQQMGRDPD**L**EQRAEE**L**ARENEE**LL**EKEAEE**L**EQENAE**L**EGECQG**L**EARNRE**L**RERAESVEREIQYVKD**LL**IEVYKARSQRTRSA, or encoding a mutant form of dn-ATF5, MASMTGGQQMGRDPD**G**EQRAEE**G**ARENEE**GG**EKEAEE**G**EQENAE**G**EGECQG**G**EARNRE**G**RERAESVEREIQYVKD**GG**IEVYKARSQRTRSA in which the indicated (bolded) leucines were replaced with glycines to inactivate leucine zipper activity. The fragments were subcloned into the BamH1 and XhoI site of pCMV-3Tag-1A (Agilent Technologies Inc, Santa Clara CA) plasmid for in frame N-terminal 3XFlag-tagged expression of dn-ATF5 or mutant dn-ATF5. Where indicated, experiments additional employed pLe-FLAG-GFP-dn-ATF5 as previously described^23^.

### Lentivirus preparation

Lentivirus were prepared in HEK293 cells by co-transfecting pLVX expression plasmids along with second generation lentiviral packaging plasmids using the calcium phosphate transfection method as previously described^38^. Lentiviral particles were collected, then concentrated using Lenti-X concentrator (#631231, Takara), resuspended in PBS, and stored at −80°C.

For lentiviral infection, 0.1 up to 5 × 10^7^ viral particles were added per cm^2^ of culture area, directly in the culture medium. The transduced cells were analyzed by qPCR, Western blot or flow cytometry after 3–5 days.

### Flow cytometry

Cells were dissociated with trypsin, and washed with cold PBS twice, then resuspended in 1 X binding buffer at a concentration of 1 × 10^6^ cells/ml. About 100 µl of the solution (1 × 10^5^ cells) were incubated with 5 µl of FITC Annexin V and 5 µl PI (#556547, BD, Franklin Lakes NJ) for 15 min at room temperature in the dark. The cell suspension was mixed with 400 µl of 1X binding buffer and analyzed by flow cytometry.

### qPCR

Cells were lysed and total RNA was purified using TRI regent (Molecular Research Center, Cincinnati OH) following the manufacturer’s protocol. 1 µg of mRNA was used for the synthesis of cDNA using the first-strand cDNA synthesis kit (Origene). qPCR was performed using FastStart SYBR Green Master Mix (Roche, Indianapolis IN) by using the following primer pairs: h-Survivin, CCACTGAGAACGAGCCAGACTT and GTATTACAGGCGTAAGCCACCG; h-18S RNA, AGTCCCTGCCCTTTGTACACA and GATCCGAGGGCCTCACTAAAC. The relative mRNAs levels for genes of interest were normalized to 18 S RNA.

### Western immunoblotting

Cells were homogenized in cell lysis buffer (#9803, Cell Signaling Technology, Danvers MA) with protease inhibitor mixture (#11836170001, Roche). Protein samples were prepared in X2 loading buffer (#161-0737, BioRad, Portland ME) with βME following the manufacturer’s instruction. Protein was loaded and separated by electrophoresis and then transferred onto PVDF membranes (Bio-Rad). Blots were probed with the following primary antibodies: rabbit anti-survivin (#2808, Cell Signaling Technology), anti-FLAG (#8146, Cell Signaling Technology), mouse anti-ACTIN (#3700, Cell Signaling Technology), rabbit anti-USP9X (#5751, Cell Signaling Technology). All band intensities were determined using ImageJ and normalized to ACTIN signal.

### Immunofluorescence

Cells were fixed for 10 min in 4% PFA, washed 3 times with PBS, and blocked with Superblock (#37515 Thermo Fisher Scientific, Waltham MA) with 0.3% Triton-X for 30 min at room temperature. The following primary antibodies were used for immunofluorescence: rabbit anti-FLAG (#14793, Cell Signaling Technology), rabbit anti-GFP (# PA5-22688, Invitrogen), rabbit anti-survivin (#2808, Cell Signaling Technology). Hoechst 33328 was used to stain nuclei. Images were acquired using a Zeiss epifluorescence microscope equipped with a digital camera and Axiovision software.

## Results

### Expression of dn-ATF5 depletes nuclear survivin expression in tumor cells prior to promotion of apoptotic cell death

**Fig. 1:**
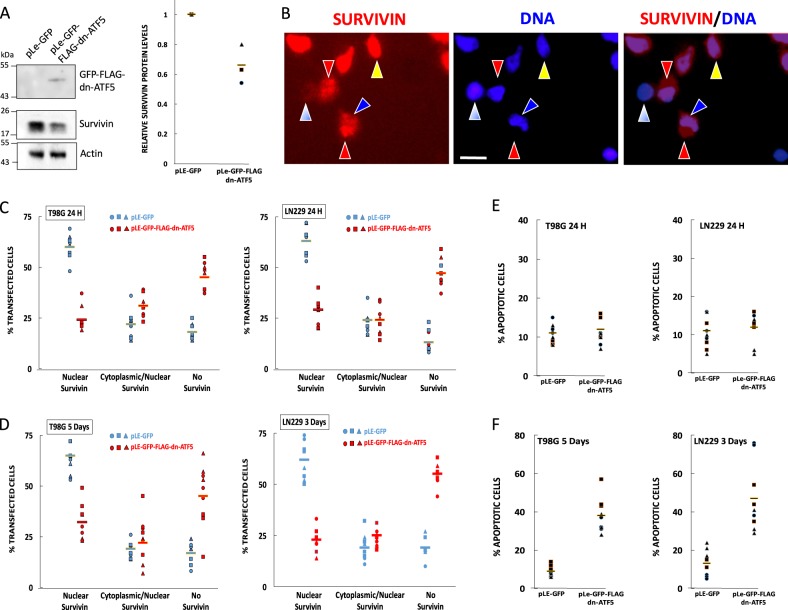
Transfection with GFP-FLAG-dn-ATF5 depletes survivin protein prior to onset of cell death. **a** Transfection with GFP-FLAG-dn-ATF5 depletes survivin in PC3 prostate tumor cells. Cells were transfected as described in methods and assessed 3 days later for relative survivin levels (normalized to ACTIN) by western immunoblotting. Left panel shows representative blot, right panel shows quantification from 3 independent experiments. **b** Examples of survivin localization in T98G cells. Transfected cultures were immunostained for survivin and co-stained with Hoechst 33828 to visualize nuclear DNA. Blue/white arrowhead shows a cell without significant survivin expression. Red and blue arrowheads show cells with both cytoplasmic (red arrowheads) and nuclear (blue arrowheads) staining. Yellow arrowhead shows a cell with nuclear-only staining. Scale bar = 10 µm. **c**, **d** Transfection with GFP-FLAG-dn-ATF5 depletes nuclear-only localized survivin and increases the proportion of cells without detectable survivin expression in T89G and LN229 cultures at 24 h (C) or at 3–5 d (D). Transfected cells (GFP+) were blindly scored for survivin localization using criteria described in panel **b**. Data are from three independent experiments, each carried out in triplicate. In each experiment about 200–300 cells were evaluated. **e** Transfection with GFP-FLAG-dn-ATF5 does not increase the incidence of apoptosis in cultures of T89G and LN229 cultures at 24 h, a time when survivin is already depleted. Transfected cells (GFP+) in cultures described in panels **c** and **d** were scored for proportion with apoptotic nuclei. Data are from three independent experiments, each carried out in triplicate. In each experiment about 200–300 cells were evaluated. **f** Transfection with GFP-FLAG-dn-ATF5 promotes cell death at 3–5 days. As in **e**, but at 5 days for T98G and 3 days for LN229 cells.

We previously reported that expression of dn-ATF5-encoding plasmids promotes apoptotic death of a wide range of tumor cell types, but not of non-transformed cells^2,3,24^. As an initial approach to assessing the potential effect of dn-ATF5 on survivin expression, we transfected PC3 prostate tumor cells with a previously described construct expressing GFP-FLAG-dn-ATF5^23^ and determined total survivin levels by western immunoblotting after 3 days (Fig. 1a). This revealed an approximate 40% loss of survivin protein expression (not accounting for a transfection efficiency of 50–70%).

Similar results were achieved by transfecting T98G glioblastoma cells with 3xFLAG-dn-ATF5 (Supplementary Fig. 1). We also carried out a parallel transfection with a mutated form of this construct in which the critical leucine residues in the leucine zipper were replaced with glycine residues. We previously found that such replacements in a dn form of ATF5 compromises its capacity to promote cell death^27^. In contrast to the non-mutated construct, the mutant construct failed to down-regulate survivin (Supplementary Fig. 1). Taken together, these findings indicate that dn-ATF5 reduces survivin expression in tumor cells and that this requires a functional leucine zipper domain.

To further assess effects of dn-ATF5 on survivin expression and localization at the cellular level, we transfected T98G and LN229 glioblastoma cells with a GFP-FLAG-dn-ATF5 construct or with a control plasmid expressing only GFP. One day after transfection, transfected (GFP+) cells were immunostained for survivin expression and blindly scored for proportions with either no, cytoplasmic/nuclear (cells with evident survivin expression in both nuclei and cytoplasm) or nuclear-only localized survivin (Fig. 1b). In both lines, about 60% of control cells showed nuclear-only localization, about 25% cytoplasmic/nuclear staining and about 15% no detectable survivin staining (Fig. 1c). In contrast, there was an approximate 3-fold increase in proportion of cells without detectable survivin expression (to almost 50% of the population) (Fig. 1c) after transfection with GFP-FLAG-dn-ATF5. This appeared to come mainly at the expense of nuclear survivin for which there was a corresponding loss in proportion of cells with immunostaining in this compartment (Fig. 1c). These effects were sustained in that they were also evident 3–5 days after transfection (Fig. 1d).

As noted above, dn-ATF5 expression triggers extensive apoptotic death of tumor cells. This raised the question as to whether survivin depletion might either precede or be a result of the death process. We therefore monitored the proportions of cells with apoptotic nuclei at 1–5 days after transfection. In agreement with prior findings^2^, this revealed no increase in apoptosis compared to controls at 24 h after transfection (Fig. 1e), a time when survivin expression was already diminished. This contrasted with later times when both loss of survivin and increased apoptotic death were apparent (Fig. 1f). Thus, survivin appears to be lost prior to the appearance of apoptotic nuclei and to be sustained during the death process.

### Cell penetrant synthetic CP-dn-ATF5 depletes survivin protein in multiple tumor cell lines

**Fig. 2:**
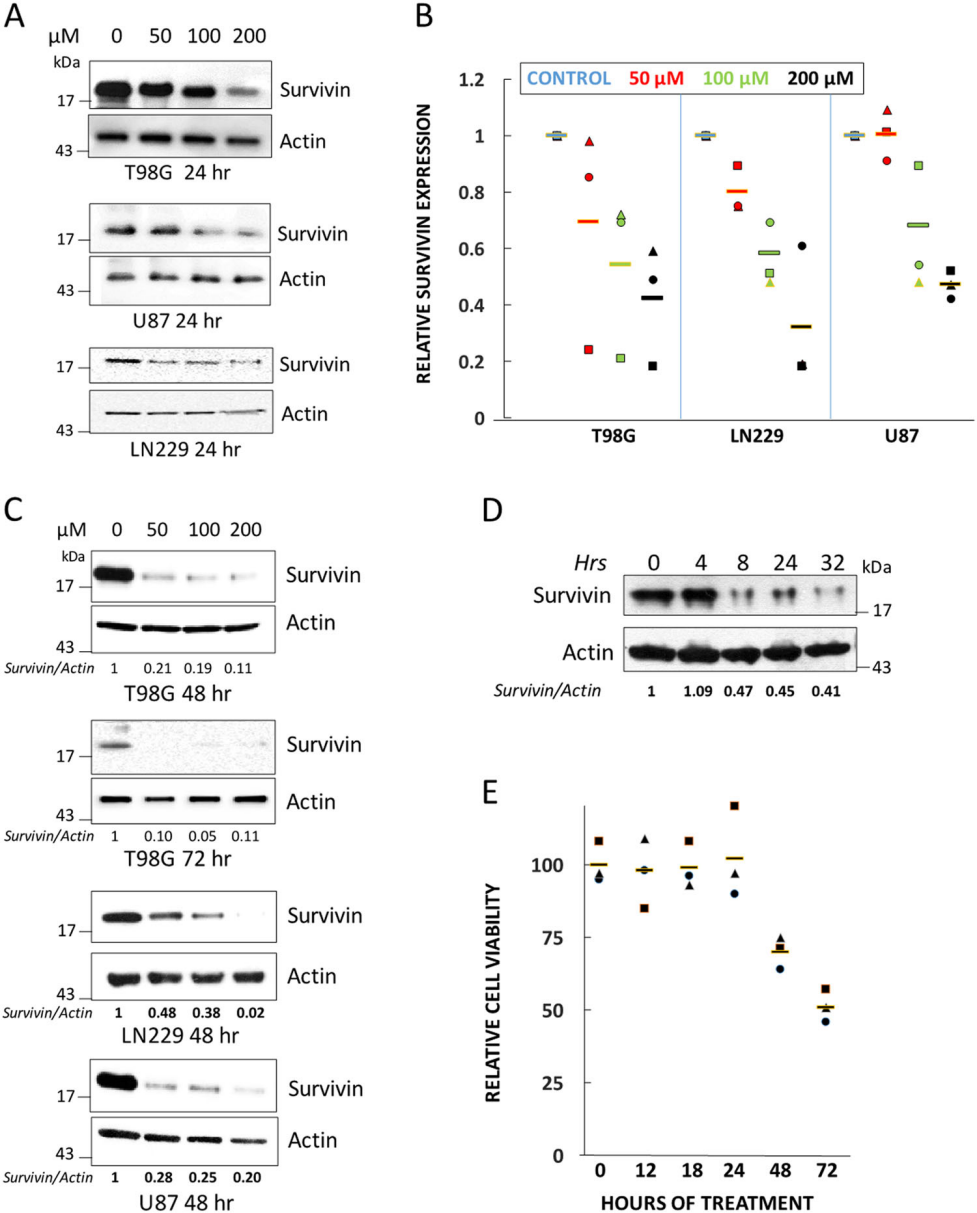
Treatment with CP-dn-ATF5 causes a dose- and time-dependent depletion of survivin protein in multiple cancer cell lines prior to loss of cell viability. **a**, **b** CP-dn-ATF5 depletes survivin in T98G, U87 and LN229 glioblastoma cell lines at 24 h after treatment. **a** Representative western blots. **b** Data are from three independent experiments. **c** CP-dn-ATF5 depletes survivin in T98G, U87 and LN229 glioblastoma cell lines at 48 and 72 h after treatment. Normalized survivin to ACTIN ratios are shown for each lane. **d** Treatment with CP-dn-ATF5 causes a rapid depletion of survivin protein in T98G cultures. Normalized survivin to ACTIN ratios are shown for each lane. **e** Cell death caused by CP-dn-ATF5 (100 µM) in T98G cultures occurs after survivin depletion. Relative numbers of cells per culture were determined at the indicated times. Three replicate cultures were evaluated at each time point

We previously described a synthetic version CP-dn-ATF5 that contains a penetratin domain that permits rapid cellular uptake of the peptide and that promotes apoptotic death of a wide variety of tumor types in vitro and in vivo^26,27^. We therefore next tested whether this potential therapeutic prototype form of dn-ATF5 would also affect tumor cell survivin protein levels. CP-dn-ATF5 has the advantage that it rapidly reaches all cells in cultures and that the effects on survivin can therefore be readily quantified. We initially treated T98G, U87 and LN229 cells with 50, 100, and 200 µM CP-dn-ATF5 for 24 h and assessed relative survivin levels. These concentrations of CP-dn-ATF5 have little effect on cell survival or numbers at 24 h, and trigger apoptotic death in a dose-responsive manner beginning at 2 days and strongly manifest at 3 days of treatment^27^. The data reveal a consistent loss of survivin protein within 24 h with some degree of variability from experiment to experiment (Fig. 2a, b).

We next applied single doses of 50, 100, and 200 µM CP-dn-ATF5 to 12 different human tumor cell lines of various origins (prostate [PC3, DU145], colon [HCT116], glioblastoma [U251, U87, T98G, LN229, and GBM12], breast [MDA-MB-231, BT474, MDA-MB-468 and MCF7] for 1–3 days and then measured the relative levels of survivin protein. Dose-dependent reductions in survivin protein were seen in all lines at 24 h and persisted for the 3 days of treatment with a range of responses, reaching survivin depletion of over 90% in some cases (Fig. 2c and Supplementary Fig. 2).

We also examined the onset time of survivin protein loss with CP-dn-ATF5 at 100 µM in T98G cultures. There was no significant effect at 4 h, but reduction of survivin levels was evident at 8 h and beyond (Fig. 2d). To determine whether this depletion of survivin might be the result of cell death, we also assessed cell viability over time and confirmed no loss of cells at 24 h with death evident only at 48 and 72 h (Fig. 2e). Taken together, our data indicate that CP-dn-ATF5 depletes survivin levels in multiple tumor lines and appears to do so well before the appearance of cell death.

### CP-dn-ATF5 depletes survivin mRNA in multiple tumor cell lines

**Fig. 3:**
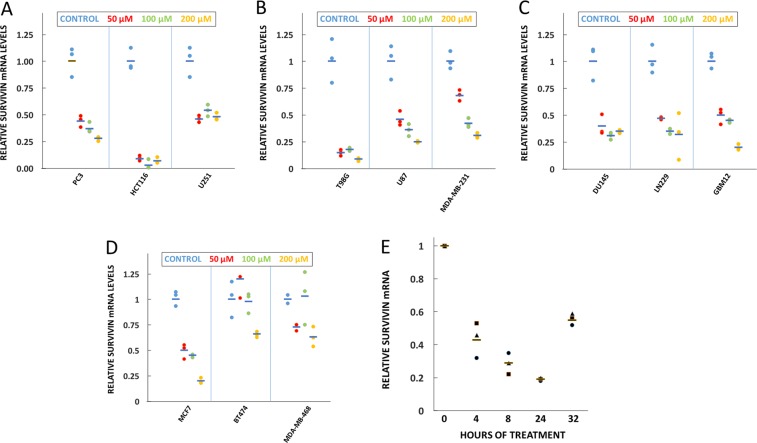
Treatment with CP-dn-ATF5 causes a dose- and time-dependent depletion of survivin mRNA in multiple cancer cell lines. **a**–**d** CP-dn-ATF5 depletes survivin mRNA in multiple cancer cell lines at 48 h of treatment. Data are from three independent experiments. **e** CP-dn-ATF5 (100 µM) causes rapid depletion of survivin mRNA in T98G cells. Values are from one experiment carried out in triplicate

To determine whether CP-dn-ATF5 affects levels of survivin mRNA as well as protein, we carried out qPCR on the 12 cell lines listed above after a single dose exposure to 50, 100, or 200 µM peptide for 48 h. In each case, there was substantial depletion of survivin mRNA, with a range of responsiveness among the lines (Fig. 3a–d). Additional analyses of survivin mRNA in multiple lines showed time and dose-dependent loss of survivin mRNA at 24 and 72 h of treatment with changes apparent within 24 h (Supplementary Figs. 3 and 4). A time course study of survivin mRNA levels in T98G cells (Fig. 3e) parallel to that described above for survivin protein (Fig. 2d) revealed that survivin transcripts were already substantially decreased by 4 h of CP-dn-ATF5 exposure. This well precedes the onset of cell death in such cells and occurs before depletion of survivin protein (Fig. 2e).

### CP-dn-ATF5 depletes virally-expressed exogenous survivin protein in multiple tumor cell lines

**Fig. 4:**
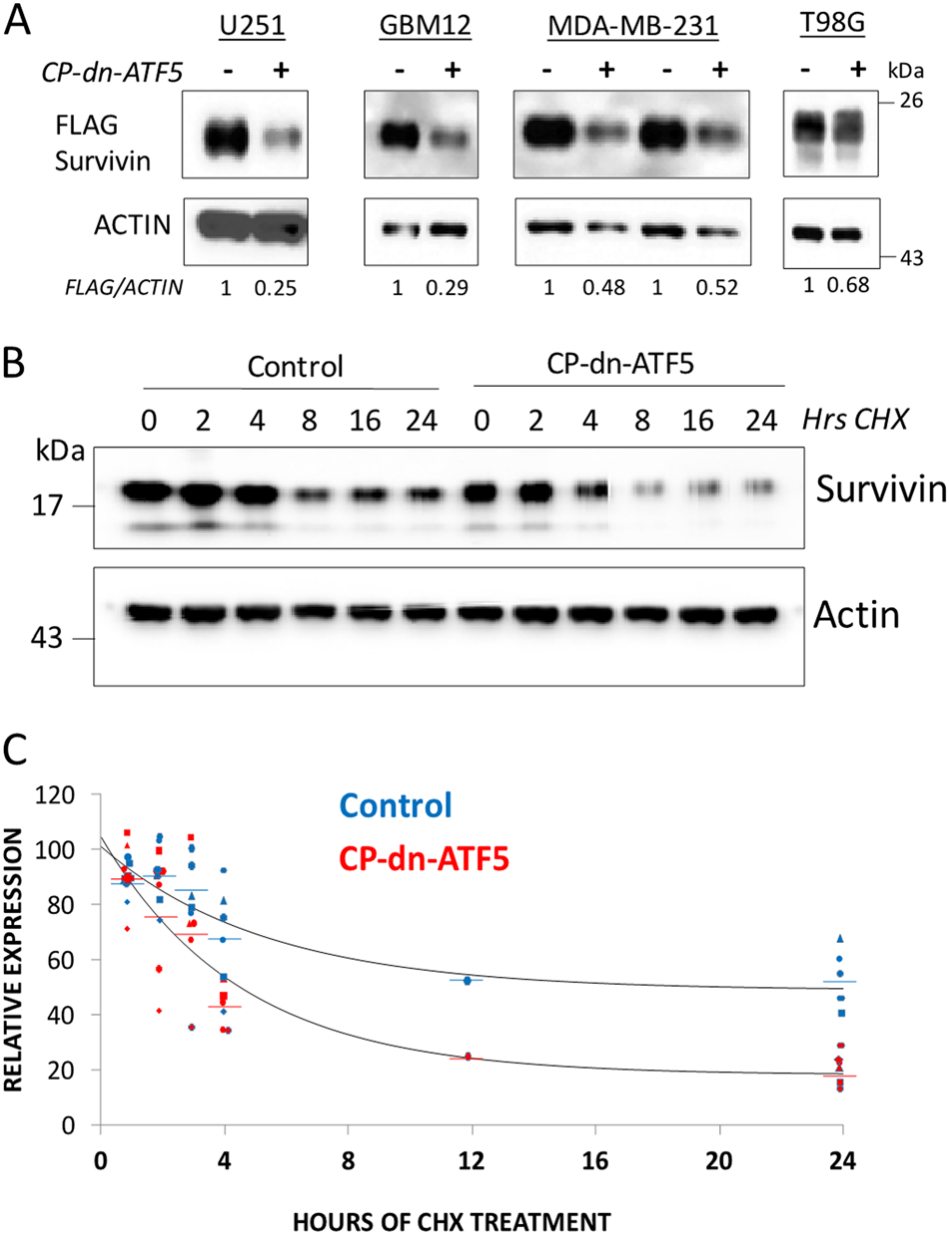
CP-dn-ATF5 promotes the turnover of survivin protein. **a** CP-dn-ATF5 decreases the expression of exogenous FLAG-survivin. Indicated cell lines were infected with lentivirus expressing FLAG-survivin and 48 h later were treated with 100 µM CP-dn-ATF5 for 72 h and then assessed for relative (to ACTIN) FLAG-survivin levels by western immunoblotting. Normalized survivin to ACTIN ratios are shown for each lane. **b** CP-dn-ATF5 accelerates the turnover of survivin protein in T98G cells. Replicate cultures were treated for 24 h with or without (Control) 100 µM CP-dn-ATF5 and then exposed to 50 µM cycloheximide for the indicated times in the continued presence or absence of CP-dn-ATF5 and then assessed for relative survivin levels by western immunoblotting. **c** CP-dn-ATF5 accelerates the turnover of survivin protein in T98G cells. Cultures were treated as in **b** and relative survivin expression determined vs ACTIN and normalized to the zero time value in each independent experiment. Numbers of independent experiments for various points are as follows: Control, 1 h, *n* = 5; 2 h, *n* = 6; 3 h, *n* = 5; 4 h, *n* = 6; 12 h, *n* = 1; 24 h, = 5. CP-dn-ATF5: 1 h, *n* = 5; 2 h, *n* = 6; 3 h, *n* = 5; 4 h, *n* = 5; 12 h, *n* = 1, 24 h, *n* = 6. Mean data points were fitted to an exponential curve

While our results indicate that CP-dn-ATF5 causes survivin mRNA depletion, the possibility remained that CP-dn-ATF5 also causes survivin protein loss by promoting its turnover. To assess this, we infected multiple tumor lines with lentivirus expressing FLAG-tagged survivin driven by a CMV promoter. Despite expression of the protein from a heterologous promoter, exposure to 100 µM CP-dn-ATF5 for 3 d caused substantial reduction in expression of the exogenous survivin protein in all lines tested (Fig. 4a). This observation suggests that CP-dn-ATF5 causes depletion of survivin protein at least partly by mechanisms independent from its effects on survivin mRNA.

### CP-dn-ATF5 destabilizes cellular survivin protein and promotes survivin loss via proteasomal degradation

We next examined whether, as appears for over-expressed survivin, CP-dn-ATF5-promoted reduction of endogenous survivin also involves a non-transcriptional mechanism. Prior reports indicate that cellular survivin levels are subject to post-transcriptional regulation by degradation^39,40^. To assess whether CP-dn-ATF5 affects survivin stability, we pretreated T98G cells with or without this peptide for 24 h and then exposed them to the translational inhibitor cycloheximide for various times before determining relative survivin protein levels by western immunoblotting. As shown in Fig. 4b, c, this revealed that CP-dn-ATF5 significantly accelerates survivin turnover under conditions when survivin synthesis is blocked. These findings thus indicate that survivin depletion caused by dn-ATF5 likely reflects decreased survivin protein stability.

**Fig. 5:**
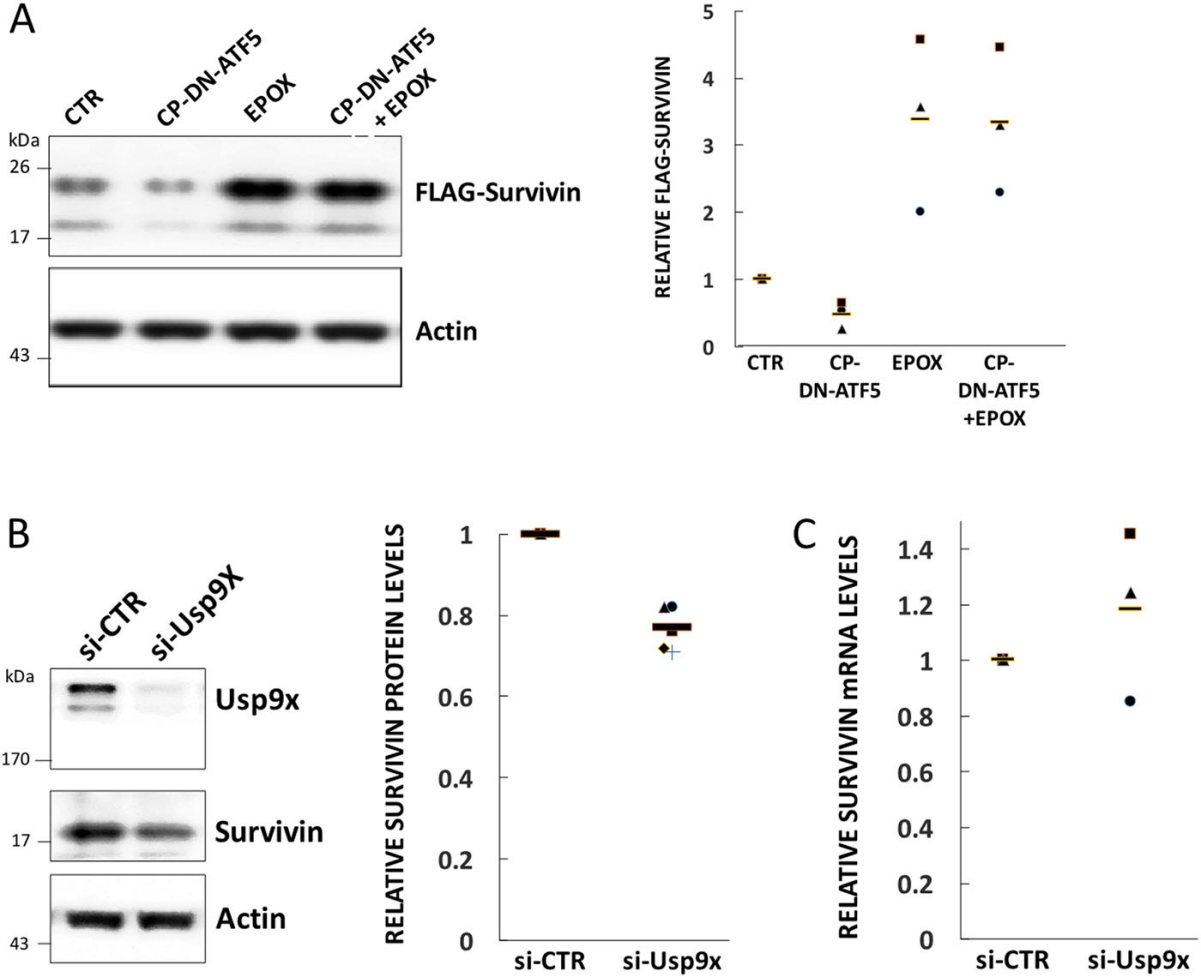
Proteasomal turnover and USP9X contribute to the depletion of survivin protein by CP-dn-ATF5. **a** Proteasomal inhibitor epoxomicin suppresses loss of survivin expression promoted by CP-dn-ATF5. T98G cells stably expressing FLAG-survivin were treated for 100 µM CP-dn-ATF5 for 24 h in presence or absence (Control, CTR) of 10 nM epoxomicin (EPOX) and assessed by western immunoblotting for levels of FLAG-survivin and ACTIN. Left panel shows representative Western immunoblot. Right panel shows quantification of relative FLAG-survivin levels under each condition. Data are from three independent experiments. **b** Knockdown of USP9X reduces levels of survivin protein in T98G cells. Cultures were transfected with control (CTR) or USP9X siRNA for 72 h and assessed 3 days later for USP9X, survivin and ACTIN protein levels by western immunoblotting (left panel). Right panel shows relative survivin protein levels for five independent experiments. **c** USP9X knockdown does not affect survivin mRNA levels in T98G cells. Cultures were treated as in **b** and assessed for relative survivin mRNA levels. Data are from 3 independent experiments

To approach the issue of the mechanism by which dn-ATF5 affects survivin stability, we generated T98G cells stably over-expressing FLAG-survivin on a heterologous promoter. To determine whether the post-translational component of the effect of CP-dn-ATF5 on survivin is mediated by the proteasome, we exposed the survivin over-expressing cells to 100 µM CP-dn-ATF5 for 24 h in presence or absence of the proteasomal inhibitor epoxomicin (Fig. 5a). Under such circumstances, FLAG-survivin levels decreased with CP-dn-ATF5 alone by about 50% compared with untreated controls; with epoxomicin alone, FLAG-survivin levels rose by about three-fold and this level was not reduced by CP-dn-ATF5 co-treatment. Taken together, these data thus indicate that CP-dn-ATF5 decreases survivin stability and support the idea that enhanced proteasomal turnover contributes to survivin depletion caused by CP-dn-ATF5.

### USP9X depletion contributes to the CP-dn-ATF5-mediated decrease in survivin stability

We next explored the mechanism by which CP-dn-ATF5 treatment leads to survivin protein destabilization. The deubiquitinase USP9X has been shown to deubiquinate survivin and thus potentially to inhibit its proteasomal degradation^41^. Although USP9X downregulation in HeLa cells was reported without effect on survivin protein levels^41^, similar downregulation of USP9X in SF188 and Panc-1 cells resulted in survivin protein depletion^42,43^. Our past work has shown that CP-dn-ATF5 dramatically reduces USP9X levels in multiple tumor cell lines including T98G cells^27^. We therefore asked whether such an effect of CP-dn-ATF5 on USP9X in T98G cells could contribute to the depletion of survivin seen with this agent. Accordingly, we used a previously described USP9X-directed siRNA^27^ to knockdown USP9X and compared survivin levels with those in cells receiving a control siRNA. This revealed a significant loss of survivin protein (Fig. 5b), but not survivin mRNA (Fig. 5c), thus indicating that the reduction of USP9X caused by CP-dn-ATF5 treatment likely contributes to survivin protein depletion.

### Is survivin depletion by CP-dn-ATF5 sufficient to account for the anti-tumor actions of this agent?

**Fig. 6:**
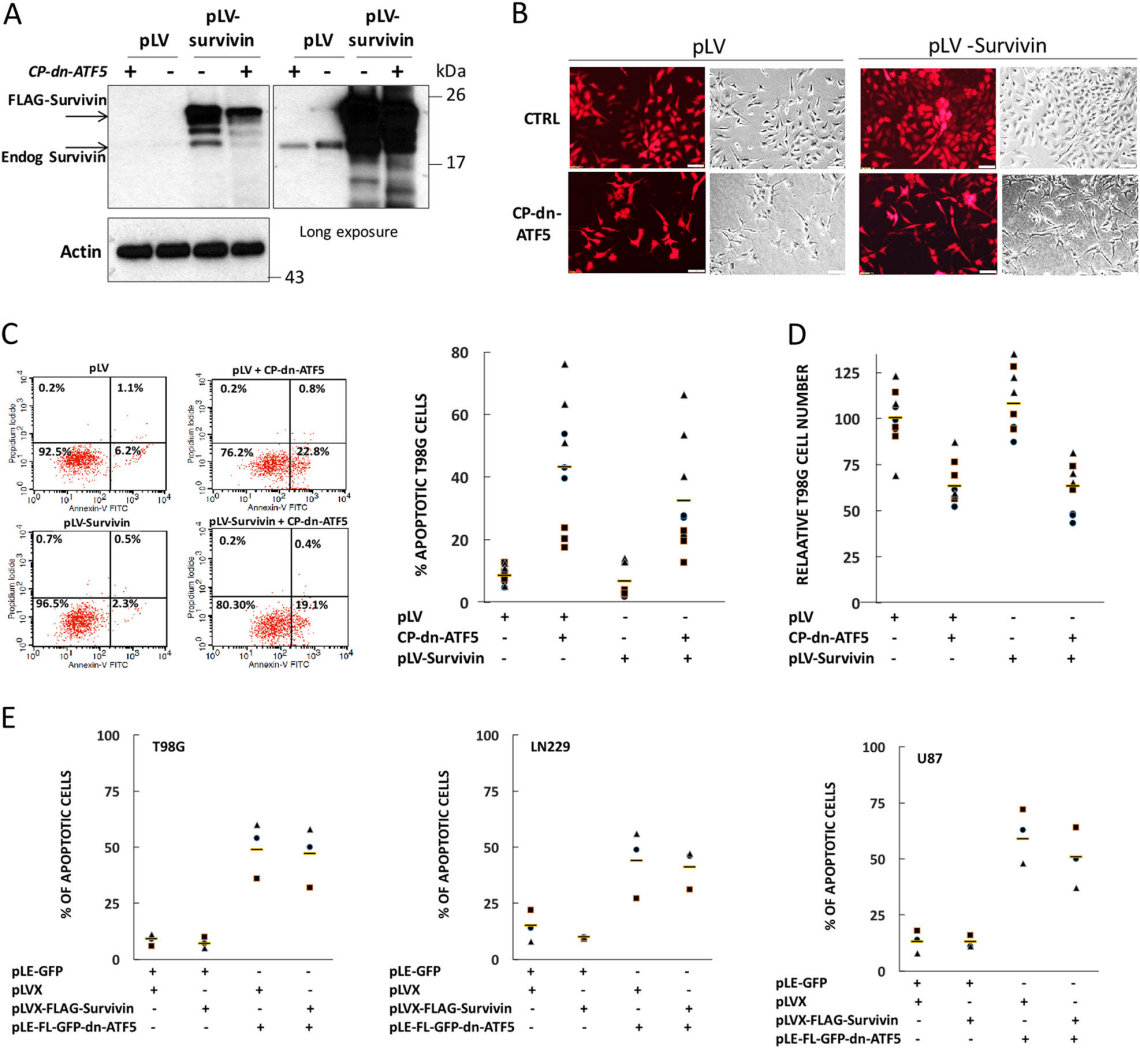
Survivin over-expression is not sufficient to rescue tumor cells from apoptotic death promoted by dn-ATF5. **a** CP-dn-ATF5 reduces expression of over-expressed FLAG-survivin, but to levels far in excess of endogenous survivin. T98G cultures were infected with lentivirus expressing either pLVX-EF1α-IRES-mCherry (pLV in Figure) or pLVX-EF1α-FLAG-survivin-IRES-mCherry (pLV-survivin in Figure) as indicated and 24 h later treated with or without 100 µM CP-dn-ATF5 for 3 d and assessed for exogenous and endogenous survivin by western immunoblotting with anti-survivin. Long exposure shows levels of endogenous survivin. **b** Survivin over-expression does not rescue the effects of CP-dn-ATF5 on number or appearance of T98G cells. Cultures were infected with lentiviruses described in panel **a** as indicated and 24 h later were treated with 100 µM CP-dn-ATF5 for 3 days. Panels show immunofluoresence for mcherry (red) or phase contrast images. Scale bars = 50 µm. **c** Survivin over-expression does not rescue T98G cells from apoptotic death promoted by CP-dn-ATF5. Cultures were infected with lentivirus described in panel **a** and 24 h later were treated with 100 µM CP-dn-ATF5 for 3 days. Cultures were harvested and analyzed for proportion of apoptotic cells by flow cytometry. Left panel shows the representative cytometry data and right panel shows quantitative results from three independent experiments, each in triplicate. **d** Survivin over-expression does not rescue cell number in T98G cultures treated with CP-dn-ATF5. Cultures were infected with above described lentiviruses and 24 h later were treated with or without 100 µM CP-dn-ATF5 as indicated for 3 days. Cultures were harvested and analyzed for total cell numbers. Data are from three independent experiments, each in triplicate. **e** Survivin over-expression does not rescue promotion of apoptosis by FLAG-GFP-dn-ATF5 in multiple cancer cell lines. Indicated cell lines were co-transfected as shown with a ratio of pLVX:pLE constructs of 3:1 and transfected (GFP+) cells were assessed for proportion with apoptotic nuclei 3 days later. Data are from three independent experiments

Numerous studies have documented that reduction of survivin levels is sufficient to promote death of various tumor cell types^30–32,34^. We confirmed this with an siRNA targeting survivin in T98G, HCT116, MCF7, and MDA-MB-468 cells that induced apoptotic death in each case (Supplementary Fig. 5). However, while direct targeting of survivin expression has been raised as a potential treatment for a variety of cancers, to this point, no successful anti-survivin monotherapy has emerged. In this context, we thus asked whether the capacity of CP-dn-ATF5 to deplete survivin is necessary for its ability to promote tumor cell death or whether this agent also has additional death-promoting activities. To address this, we first over-expressed FLAG-survivin in T98G tumor cells and then assessed the capacity of CP-dn-ATF5 to promote their death. As shown in Fig. 6a, although CP-dn-ATF5 reduced the expression of the FLAG-survivin as anticipated, even under these conditions exogenous survivin was expressed at far higher levels than the corresponding endogenous protein. Despite such high survivin over-expression, it failed to significantly protect T98G cells from death caused by 3 days of CP-dn-ATF5 treatment as indicated by morphology (Fig. 6b), percentage of apoptotic cells as determined by flow analysis (6 C), or cell numbers (6D). We also observed similar results by flow analysis of U87 cells and by cell counts of LN229, GBM12, MDA-MB-231, U251, and DU145 cultures transfected to over-express survivin (Supplementary Figs. 6, 7). Finally, to control for the possibility that CP-dn-ATF5 kills survivin over-expressing tumor cells by a mechanism apart from its dn-ATF5 activity, we also asked whether survivin over-expression would rescue cells transfected with the pLe-GFP-FLAG-dn-ATF5 plasmid. As shown in Fig. 6e, over-expressed survivin also failed to protect from plasmid-delivered dn-ATF5. These findings thus support the conclusion that while CP-dn-ATF5 rapidly depletes cells of survivin, it has additional actions that can promote cell death that cannot be rescued by survivin over-expression.

The inability of survivin over-expression to rescue tumor cells from the lethal effects of dn-ATF5 exposure raised the question of whether CP-dn-ATF5 might kill tumor cells by a non-apoptotic mechanism. Our past work showed that tumor cell death promoted by transfected dn-ATF5 plasmid is apoptotic in nature and is blocked by inhibition of caspase activity^2^. We confirmed this for CP-dn-ATF5 in the present study in which the caspase inhibitor zVAD rescued cell numbers and the occurrence of apoptotic nuclei from the effects of 3 days of peptide exposure (Supplementary Fig. 8). Past work has shown that dn-ATF5 expression leads to downregulation of pro-survival BCL2^9,27,28^. We therefore additionally assessed whether BCL2 over-expression would protect T98G cells from 3 days of CP-dn-ATF5 treatment. While Bcl2 over-expression reduced the presence of apoptotic nuclei, such protection from CP-dn-ATF5 was only partial (Supplementary Fig. 9). Taken together, these findings support the idea that dn-ATF5 promotes caspase-dependent apoptotic death of cancer cells and that it does so by multiple pro-apoptotic actions including downregulation of survivin and BCL2. Moreover, owing to the multiplicity of these actions, replacement of a single pro-survival protein such as survivin is insufficient to maintain viability.

## Discussion

The present findings establish that dn-ATF5 consistently depletes survivin expression in a wide variety of human tumor cell lines. This appears to occur by both decreases in levels of survivin mRNA and enhanced survivin turnover. These decreases occur rapidly and are durable. Given survivin’s roles in cancer cell proliferation, survival, metastasis, and therapeutic resistance^30–32,34,35^, dn-ATF5-induced survivin depletion is highly likely to contribute to dn-ATF5’s anti-tumor actions.

We observed that both plasmid-encoded and a penetratin-linked synthetic forms of dn-ATF5-promoted survivin depletion. The plasmid-encoded form has N-terminal GFP and FLAG tags followed by a linker region and retains the 25 amino acids C-terminal to the leucine zipper domain of ATF5. These sequences are not present in CP-dn-ATF5^27^, thus indicating that they are neither required for, nor interfere with, the capacity of dn-ATF5 to deplete survivin. Likewise, addition of the N-terminal penetratin domain in the synthetic form does not appear to interfere with its capacity to regulate survivin expression.

It was also conceivable that plasma-encoded and penetratin-linked dn-ATF5 might be present in different subcellular compartments that would result in distinct effects on survivin levels. This did not appear to be the case, indicating that both forms have similar activities. We also evaluated a form of 3xFLAG-dn-ATF5 mutated in the leucine zipper domain. This mutant form failed to deplete survivin, demonstrating that leucine zipper function is indispensable for dn-ATF5’s capacity to affect survivin expression.

One potential issue about the effects of dn-ATF5 on survivin expression was whether this was a response to, rather than contributor to, apoptotic death. Our findings with both transfected and penetratin-linked dn-ATF5 showed depletion of survivin mRNA and protein well before onset of cell death, thus supporting a role for survivin depletion as a primary contributor rather than responder to cell death triggered by dn-ATF5.

Our findings indicate that dn-ATF5 selectively depletes nuclear-localized survivin. It has been reported that survivin turns over more efficiently in nuclei^44–46^. In this light, given that dn-ATF5 rapidly diminishes survivin mRNA levels, loss of nuclear survivin may reflect decreased survivin synthesis that cannot keep up with the level of degradation in this compartment. Additionally, or alternatively, dn-ATF5 may affect survivin movement between the cytoplasmic and nuclear compartments, so that nuclear import is reduced or export is increased. In either case, loss of nuclear localization would suggest that at least one consequence of survivin depletion by dn-ATF5 is defective cell cycle progression.

**Fig. 7:**
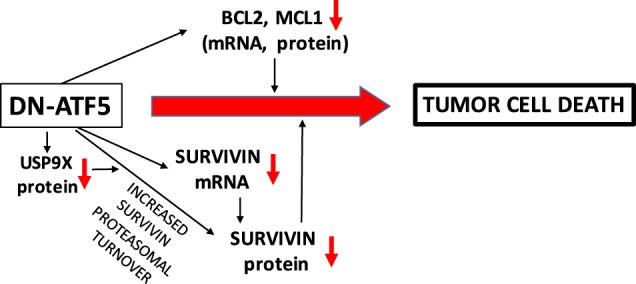
Proposed role of survivin regulation in the mechanism by which dn-ATF5 kills tumor cells

The mechanisms that underlie the depletion of survivin by dn-ATF5 appear to be complex with both reduction of mRNA levels and post-translational degradation playing roles (Fig. 7). It is presently unknown whether survivin is a direct or indirect transcriptional target of ATF5 or whether dn-ATF5 might act by binding and sequestering another partner that influences survivin mRNA or protein expression. Our findings that dn-ATF5 reduces expression of survivin protein encoded by plasmids with heterologous promoters and that it accelerates turnover of endogenous survivin establish a role for a post-translational mechanism in survivin depletion. Prior work shows that survivin is subject to proteasomal degradation^39,40^. Consistent with this, we found that the proteasomal inhibitor epoxomicin elevated expression of transfected FLAG-survivin. We also observed that epoxomicin blocked the depletion of FLAG-survivin by CP-dn-ATF5, a finding that supports the idea that one mechanism by which dn-ATF5 reduces survivin is by promoting its proteasomal destruction. Survivin appears to be protected from proteasomal degradation by interaction with partners such as HSP90^47^ and by deubiquitination by enzymes including USP9X^42,43^. We previously reported that CP-dn-ATF5 substantially reduces expression of USP9X in a variety of tumor cell lines^27^. This raised the possibility that enhanced survivin turnover by dn-ATF5 is mediated at least in part by USP9X depletion (Fig. 7) and in support of this, we found that USP9X knockdown in T98G cells results in a significant reduction of survivin protein levels.

Among the issues explored here was whether survivin depletion fully accounts for the apoptotic activity of dn-ATF5. The degree of survivin depletion in many cell lines achieved with dn-ATF5 (in some cases, >90%) was on the order reached by survivin si- or sh-RNA treatments that elicit apoptotic tumor cell death. Thus, it appears that the depletion of survivin caused by dn-ATF5 is likely to be sufficient to cause death. On the other hand, our experiments indicated that death of tumor cells promoted by CP-dn-ATF5 was not rescued by survivin over-expression. This suggests that dn-ATF5 triggers pro-apoptotic events in addition to survivin depletion that are sufficient to kill cancer cells. Thus far, additional identified actions of dn-ATF5 that could contribute to its apoptotic actions include depletion of MCL1 and BCL2^5,10,27,28^ (Fig. 7) and it is highly likely that more remain to be identified. The potential capacity of dn-ATF5 therapeutics to deplete survivin as well as other survival-promoting proteins may represent an advantage over drugs such as siRNAs that target only survivin.

In addition to promoting cancer cell death as monotherapies, agents that reduce survivin expression or activity are reported to sensitize tumor cells to chemotherapeutics or radiation^30–35^. Because dn-ATF5 depletes survivin in tumor cells, this suggests that it may also serve in combination therapies to overcome therapeutic resistance. This possibility is supported by reports that dn-ATF5 sensitizes pancreatic cancer cells to paclitaxel^9^ and that ATF5 promotes radioresistance of lung cancer cells^11^.

## Supplementary information

Supplementary figure 1

Supplementary Fig 2

Supplementary Figure 3

Supplementary Fig 4

Supplementary Figure 5

Supplementary Figure 6

Supplementary Figure 7

Supplementary Fig 8

Supplementary Fig 9

Supplementary figure legends

## References

[1] Dong S (2005). Histology-based expression profiling yields novel prognostic markers in human glioblastoma. J. Neuropathol. Exp. Neurol..

[2] Angelastro JM (2006). Selective destruction of glioblastoma cells by interference with the activity or expression of ATF5. Oncogene.

[3] Monaco SE, Angelastro JM, Szabolcs M, Greene LA (2007). The transcription factor ATF5 is widely expressed in carcinomas, and interference with its function selectively kills neoplastic, but not nontransformed, breast cell lines. Int. J. Cancer.

[4] Greene LA, Lee HY, Angelastro JM (2009). The transcription factor ATF5: role in neurodevelopment and neural tumors. J. Neurochem..

[5] Sheng Z (2010). A genome-wide RNA interference screen reveals an essential CREB3L2-ATF5-MCL1 survival pathway in malignant glioma with therapeutic implications. Nat. Med..

[6] Sheng Z, Evans SK, Green MR (2010). An activating transcription factor 5-mediated survival pathway as a target for cancer therapy?. Oncotarget.

[7] Li G, Xu Y, Guan D, Liu Z, Liu DX (2011). HSP70 protein promotes survival of C6 and U87 glioma cells by inhibition of ATF5 degradation. J. Biol. Chem..

[8] KONG XIANGHENG, MENG WENJIAN, ZHOU ZONGGUANG, LI YUAN, ZHOU BIN, WANG RONG, ZHAN LAN (2011). Overexpression of activating transcription factor 5 in human rectal cancer. Experimental and Therapeutic Medicine.

[9] Hu M (2012). Interference with ATF5 function enhances the sensitivity of human pancreatic cancer cells to paclitaxel-induced apoptosis. Anticancer Res..

[10] Chen A (2012). ATF5 is overexpressed in epithelial ovarian carcinomas and interference with its function increases apoptosis through the downregulation of Bcl-2 in SKOV-3 cells. Int. J. Gynecol. Pathol..

[11] Ishihara S (2015). Activating transcription factor 5 enhances radioresistance and malignancy in cancer cells. Oncotarget.

[12] Nukuda Akihiro, Endoh Hiroki, Yasuda Motoaki, Mizutani Takeomi, Kawabata Kazushige, Haga Hisashi (2016). Role of ATF5 in the invasive potential of diverse human cancer cell lines. Biochemical and Biophysical Research Communications.

[13] Angelastro James M. (2017). Targeting ATF5 in Cancer. Trends in Cancer.

[14] Wang Mengyuan, Hu Ming, Li Zhaohua, Qian Dongmeng, Wang Bin, Liu David X. (2017). miR-141-3p functions as a tumor suppressor modulating activating transcription factor 5 in glioma. Biochemical and Biophysical Research Communications.

[15] Deng Pan, Haynes Cole M. (2017). Mitochondrial dysfunction in cancer: Potential roles of ATF5 and the mitochondrial UPR. Seminars in Cancer Biology.

[16] Hu M (2017). Human cytomegalovirus immediate-early protein promotes survival of glioma cells through interacting and acetylating ATF5. Oncotarget.

[17] Ben-Shmuel, S. et al. Activating Transcription Factor-5 Knockdown Reduces Aggressiveness of Mammary Tumor Cells and Attenuates Mammary Tumor Growth. *Front. Endocrinol*. (Lausanne) **8**, 173, 10.3389/fendo.2017.00173 (2017). 10.3389/fendo.2017.00173 PMC5519529 28785242

[18] Feldheim J (2018). Expression of activating transcription factor 5 (ATF5) is increased in astrocytomas of different WHO grades and correlates with survival of glioblastoma patients. Onco Targets Ther..

[19] Hansen MB (2002). Mouse Atf5: molecular cloning of two novel mRNAs, genomic organization, and odorant sensory neuron localization. Genomics.

[20] Al Sarraj, J., Vinson, C. & Thiel, G. Regulation of asparagine synthetase gene transcription by the basic region leucine zipper transcription factors ATF5 and CHOP. *Biol. Chem*. 386, 873–879, 10.1515/BC.2005.102 (2005). 10.1515/BC.2005.102 16164412

[21] Potapov V, Kaplan JB, Keating AE (2015). Data-driven prediction and design of bZIP coiled-coil interactions. PLoS Comput. Biol..

[22] Sears TK, Angelastro JM (2017). The transcription factor ATF5: role in cellular differentiation, stress responses, and cancer. Oncotarget.

[23] Angelastro JM (2003). Regulated expression of ATF5 is required for the progression of neural progenitor cells to neurons. J. Neurosci..

[24] Arias A (2012). Regulated ATF5 loss-of-function in adult mice blocks formation and causes regression/eradication of gliomas. Oncogene.

[25] Dupont E, Prochiantz A, Joliot A (2015). Penetratin story: an overview. Methods Mol. Biol..

[26] Cates CC (2016). Regression/eradication of gliomas in mice by a systemically-deliverable ATF5 dominant-negative peptide. Oncotarget.

[27] Karpel-Massler G (2016). A synthetic cell-penetrating dominant-negative ATF5 peptide exerts anticancer activity against a broad spectrum of treatment-resistant cancers. Clin. Cancer Res..

[28] Dluzen D, Li G, Tacelosky D, Moreau M, Liu DX (2011). BCL-2 is a downstream target of ATF5 that mediates the prosurvival function of ATF5 in a cell type-dependent manner. J. Biol. Chem..

[29] Ambrosini G, Adida C, Altieri DC (1997). A novel anti-apoptosis gene, survivin, expressed in cancer and lymphoma. Nat. Med..

[30] Altieri DC (2013). Targeting survivin in cancer. Cancer Lett..

[31] Altieri DC (2015). Survivin—The inconvenient IAP. Semin-. Cell Dev. Biol..

[32] Cheung CH (2013). Survivin—biology and potential as a therapeutic target in oncology. Onco Targets Ther..

[33] Athanasoula Kalliopi Ch., Gogas Helen, Polonifi Katerina, Vaiopoulos Aristeidis G., Polyzos Aristidis, Mantzourani Marina (2014). Survivin beyond physiology: Orchestration of multistep carcinogenesis and therapeutic potentials. Cancer Letters.

[34] Peery Robert C., Liu Jing-Yuan, Zhang Jian-Ting (2017). Targeting survivin for therapeutic discovery: past, present, and future promises. Drug Discovery Today.

[35] Li, D., Hu, C. & Li, H. Survivin as a novel target protein for reducing the proliferation of cancer cells. *Biomed. Rep*. **8**, 399–406, 10.3892/br.2018.1077 (2018). 10.3892/br.2018.1077 PMC5920485 29725522

[36] Martinez-Garcia D, Manero-Ruperez N, Quesada R, Korrodi-Gregorio L, Soto-Cerrato V (2018). Therapeutic strategies involving survivin inhibition in cancer. Med. Res. Rev.

[37] Wang NS, Unkila MT, Reineks EZ, Distelhorst CW (2001). Transient expression of wild-type or mitochondrially targeted Bcl-2 induces apoptosis, whereas transient expression of endoplasmic reticulum-targeted Bcl-2 is protective against Bax-induced cell death. J. Biol. Chem..

[38] Aime, P., Sun, X., Greene, L.A. *Gene Expression and Regulation in Mammalian Cells* Ch. 11*.* (IntechOpen, London, 2018).

[39] Zhao J, Tenev T, Martins LM, Downward J, Lemoine NR (2000). The ubiquitin-proteasome pathway regulates survivin degradation in a cell cycle-dependent manner. J. Cell Sci..

[40] Liu Y (2015). The proapoptotic F-box protein Fbxl7 regulates mitochondrial function by mediating the ubiquitylation and proteasomal degradation of survivin. J. Biol. Chem..

[41] Vong QP, Cao K, Li HY, Iglesias PA, Zheng Y (2005). Chromosome alignment and segregation regulated by ubiquitination of survivin. Science.

[42] Karpel-Massler G (2016). Inhibition of deubiquitinases primes glioblastoma cells to apoptosis in vitro and in vivo. Oncotarget.

[43] Liu, L. et al. Deubiquitinase USP9X promotes cell migration, invasion and inhibits apoptosis of human pancreatic cancer. *Oncol. Rep*. **38**, 3531–3537, 10.3892/or.2017.6050 (2017). 10.3892/or.2017.6050 29130109

[44] Connell CM, Colnaghi R, Wheatley SP (2008). Nuclear survivin has reduced stability and is not cytoprotective. J. Biol. Chem..

[45] Chan K-S, Wong C-H, Huang Y-F, Li H-Y (2010). Survivin withdrawal by nuclear export failure as a physiological switch to commit cells to apoptosis. Cell Death & Disease.

[46] Aljaberi Aysha M, Webster Jamie RM, Wheatley Sally P (2015). Mitotic activity of survivin is regulated by acetylation at K129. Cell Cycle.

[47] Fortugno P (2003). Regulation of survivin function by Hsp90. Proc. Natl Acad. Sci. USA.

