# Supplementary material for: Dominant-negative ATF5 rapidly depletes survivin in tumor cells
Source: Cell Death Dis. 2019 Sep 24;10(10):709. doi: 10.1038/s41419-019-1872-y (PMC6760124; doi:10.1038/s41419-019-1872-y)
Supplement: Supplementary file 1 — Supplementary figure 1 [file 41419_2019_1872_MOESM1_ESM.docx]

**Supplementary Fig. 1: Survivin depletion by dn-ATF5 is dependent on an intact leucine zipper domain**. T98G cells were transfected as indicated with either FLAG-DN-ATF5 or FLAG-DN-ATF5 Mutant (Mutated with the L to G substitutions listed in Methods and assessed 3 days later for relative survivin protein levels (normalized to actin) by western immunoblotting. Left panel shows a representative blot, right panel shows relative survivin protein levels for 3 independent experiments.
