# Supplementary material for: Dominant-negative ATF5 rapidly depletes survivin in tumor cells
Source: Cell Death Dis. 2019 Sep 24;10(10):709. doi: 10.1038/s41419-019-1872-y (PMC6760124; doi:10.1038/s41419-019-1872-y)
Supplement: Supplementary file 2 — Supplementary Fig 2 [file 41419_2019_1872_MOESM2_ESM.pdf]

Supplementary Figure 2

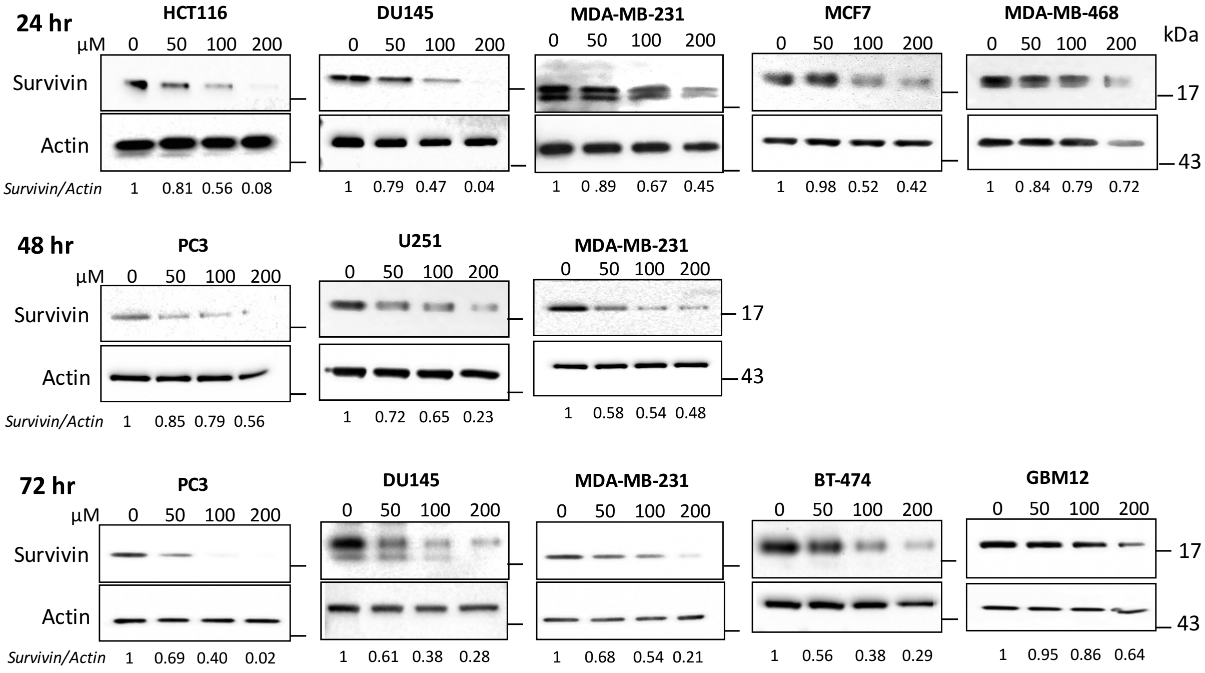


**Supplementary Fig. 2: CP-dn-ATF5 causes a persistent dose-dependent depletion of survivin protein in multiple cancer cell lines.** The indicated cell lines were treated with the CP-dn-ATF5 for 24, 48 or 72 h as indicated and then subjected to western immunoblotting to determine the expression of survivin protein relative to ACTIN. Normalized survivin to ACTIN ratios are shown for each lane.
