# Supplementary material for: Dominant-negative ATF5 rapidly depletes survivin in tumor cells
Source: Cell Death Dis. 2019 Sep 24;10(10):709. doi: 10.1038/s41419-019-1872-y (PMC6760124; doi:10.1038/s41419-019-1872-y)
Supplement: Supplementary file 3 — Supplementary Figure 3 [file 41419_2019_1872_MOESM3_ESM.docx]

**Supplementary Fig. 3: CP-dn-ATF5 depletes survivin mRNA in multiple cancer cell lines at 24 h of treatment**. **A-C**. Data are for 3 replicate cultures at each concentration for each cell line.
