# Supplementary material for: Dominant-negative ATF5 rapidly depletes survivin in tumor cells
Source: Cell Death Dis. 2019 Sep 24;10(10):709. doi: 10.1038/s41419-019-1872-y (PMC6760124; doi:10.1038/s41419-019-1872-y)
Supplement: Supplementary file 4 — Supplementary Fig 4 [file 41419_2019_1872_MOESM4_ESM.docx]

**Supplementary Fig. 4:** **CP-dn-ATF5 causes sustained depletion (72 h) of survivin mRNA in multiple cancer cell lines**. **A-D.** Data are for 3 replicate cultures at each concentration for each cell line.
