# Supplementary material for: Dominant-negative ATF5 rapidly depletes survivin in tumor cells
Source: Cell Death Dis. 2019 Sep 24;10(10):709. doi: 10.1038/s41419-019-1872-y (PMC6760124; doi:10.1038/s41419-019-1872-y)
Supplement: Supplementary file 5 — Supplementary Figure 5 [file 41419_2019_1872_MOESM5_ESM.docx]

**Supplementary Fig. 5: Knockdown of survivin causes loss of viability in T98G, HCT116, MCF7 and MDA-MB-468 cultures.** **A-C**. Replicate T98G cultures were transfected with Control (CTR) or survivin siRNA and cultured for 3 d and then replicate cultures were assessed for relative survivin protein (A,B) and cell numbers (C). Data are from 3 independent experiments. **D-H**. Replicate T98G, HCT116, MCF7 and MDA-MB-468 cultures were transfected with Control (CTR) or survivin siRNA and cultured for 3 d, and then assessed for relative expression of survivin protein (D) or for proportion of apoptotic cells by flow cytometry (E-H).
