# Supplementary material for: Dominant-negative ATF5 rapidly depletes survivin in tumor cells
Source: Cell Death Dis. 2019 Sep 24;10(10):709. doi: 10.1038/s41419-019-1872-y (PMC6760124; doi:10.1038/s41419-019-1872-y)
Supplement: Supplementary file 6 — Supplementary Figure 6 [file 41419_2019_1872_MOESM6_ESM.docx]

**Supplementary Fig. 6: Survivin over-expression does not rescue U87 cells from apoptotic death promoted by CP-dn-ATF5**. Cultures were infected with lentivirus expressing FLAG-survivin and 24 hours later were treated with 100 µM CP-dn-ATF5 for 3 d. Cultures were then harvested and analyzed for proportion of apoptotic cells by flow cytometry. Data are from 3 replicate cultures.
