# Supplementary material for: Dominant-negative ATF5 rapidly depletes survivin in tumor cells
Source: Cell Death Dis. 2019 Sep 24;10(10):709. doi: 10.1038/s41419-019-1872-y (PMC6760124; doi:10.1038/s41419-019-1872-y)
Supplement: Supplementary file 7 — Supplementary Figure 7 [file 41419_2019_1872_MOESM7_ESM.docx]

**Supplementary Fig. 7: Survivin over-expression does not rescue cell number in multiple tumor cell lines treated with CP-dn-ATF5**. Replicate cultures were infected with lentivirus expressing FLAG-survivin and 24 h later were treated with or without 100 µM CP-dn-ATF5 as indicated for 3 days. Cultures were then harvested and analyzed for total cell numbers. Data are from 3 replicate cultures for each tumor cell line.
