# Supplementary material for: Dominant-negative ATF5 rapidly depletes survivin in tumor cells
Source: Cell Death Dis. 2019 Sep 24;10(10):709. doi: 10.1038/s41419-019-1872-y (PMC6760124; doi:10.1038/s41419-019-1872-y)
Supplement: Supplementary file 8 — Supplementary Fig 8 [file 41419_2019_1872_MOESM8_ESM.pdf]

Supplementary Fig 8

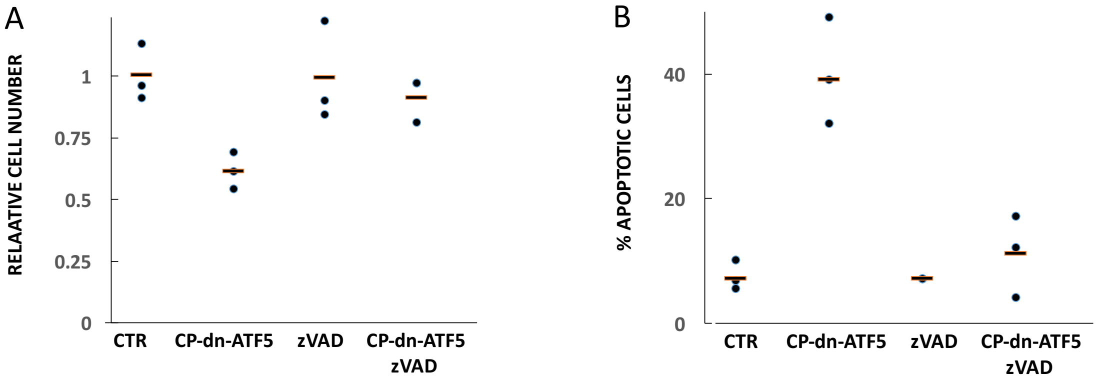

Supplementary Fig. 8: A pan-caspase inhibitor blocks the effects of CP-dn-ATF5 on growth and survival of T98G glioblastoma cells. A,B. T98G cells were treated with 100 µM CP-dn-ATF5 and 10 µM zVAD as indicated 3 d and then evaluated for relative cell number (A) and % of cells with apoptotic nuclei (B). Data are from 3 replicate cultures.
