# Supplementary material for: Dominant-negative ATF5 rapidly depletes survivin in tumor cells
Source: Cell Death Dis. 2019 Sep 24;10(10):709. doi: 10.1038/s41419-019-1872-y (PMC6760124; doi:10.1038/s41419-019-1872-y)
Supplement: Supplementary file 9 — Supplementary Fig 9 [file 41419_2019_1872_MOESM9_ESM.pdf]

Supplementary Fig 9

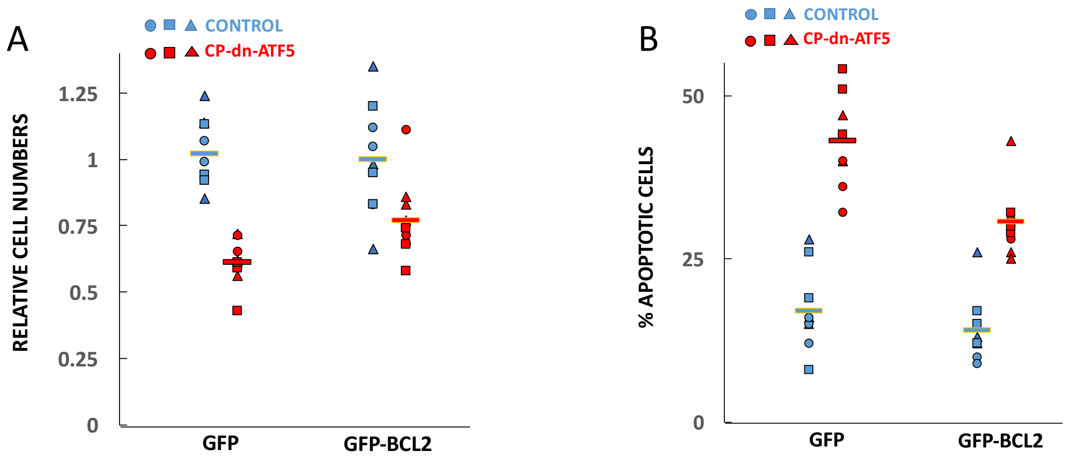

Supplementary Fig. 9: Bcl2 over-expression provides partial protection from CP-dn-ATF5 treatment. A,B. T98G cells were transfected with either GFP or GFP-BCL2 for 1 d and then exposed to 100 µM CP-dn-ATF5 for 3 additional d. The cells were then stained with Hoescht 33328 to visualize nuclei and immunostained for GFP expression and assessed for (A) relative numbers of surviving GFP+ cells (in each case normalized to its respective control) or (B) % of GFP+ cells with apoptotic nuclei. Data are from 3 independent experiments, each carried out in triplicate.
