# Supplementary material for: Dominant-negative ATF5 rapidly depletes survivin in tumor cells
Source: Cell Death Dis. 2019 Sep 24;10(10):709. doi: 10.1038/s41419-019-1872-y (PMC6760124; doi:10.1038/s41419-019-1872-y)
Supplement: Supplementary file 10 — Supplementary figure legends [file 41419_2019_1872_MOESM10_ESM.doc]

Legends to supplementary Figures:

**Supplementary Fig. 1: Survivin depletion by dn-ATF5 is dependent on an intact leucine zipper domain**. T98G cells were transfected as indicated with either FLAG-DN-ATF5 or FLAG-DN-ATF5 Mutant (Mutated with the L to G substitutions listed in Methods and assessed 3 days later for relative survivin protein levels (normalized to actin) by western immunoblotting. Left panel shows a representative blot, right panel shows relative survivin protein levels for 3 independent experiments.

**Supplementary Fig. 2: CP-dn-ATF5 causes a persistent dose-dependent depletion of survivin protein in multiple cancer cell lines.** The indicated cell lines were treated with the CP-dn-ATF5 for 24, 48 or 72 h as indicated and then subjected to western immunoblotting to determine the expression of survivin protein relative to ACTIN. Normalized survivin to ACTIN ratios are shown for each lane.

**Supplementary Fig. 3: CP-dn-ATF5 depletes survivin mRNA in multiple cancer cell lines at 24 h of treatment**. **A-C**. Data are for 3 replicate cultures at each concentration for each cell line.

**Supplementary Fig. 4:** **CP-dn-ATF5 causes sustained depletion (72 h) of survivin mRNA in multiple cancer cell lines**. **A-D.** Data are for 3 replicate cultures at each concentration for each cell line.

**Supplementary Fig. 5: Knockdown of survivin causes loss of viability in T98G, HCT116, MCF7 and MDA-MB-468 cultures.** **A-C**. Replicate T98G cultures were transfected with Control (CTR) or survivin siRNA and cultured for 3 d and then replicate cultures were assessed for relative survivin protein (A,B) and cell numbers (C). Data are from 3 independent experiments. **D-H**. Replicate T98G, HCT116, MCF7 and MDA-MB-468 cultures were transfected with Control (CTR) or survivin siRNA and cultured for 3 d, and then assessed for relative expression of survivin protein (D) or for proportion of apoptotic cells by flow cytometry (E-H).

**Supplementary Fig. 6: Survivin over-expression does not rescue U87 cells from apoptotic death promoted by CP-dn-ATF5**. Cultures were infected with lentivirus expressing FLAG-survivin and 24 hours later were treated with 100 µM CP-dn-ATF5 for 3 d. Cultures were then harvested and analyzed for proportion of apoptotic cells by flow cytometry. Data are from 3 replicate cultures.

**Supplementary Fig. 7: Survivin over-expression does not rescue cell number in multiple tumor cell lines treated with CP-dn-ATF5**. Replicate cultures were infected with lentivirus expressing FLAG-survivin and 24 h later were treated with or without 100 µM CP-dn-ATF5 as indicated for 3 days. Cultures were then harvested and analyzed for total cell numbers. Data are from 3 replicate cultures for each tumor cell line.

**Supplementary Fig. 8: A pan-caspase inhibitor blocks the effects of CP-dn-ATF5 on growth and survival of T98G glioblastoma cells**. **A,B**. T98G cells were treated with 100 µM CP-dn-ATF5 and 10 µM zVAD as indicated 3 d and then evaluated for relative cell number (A) and % of cells with apoptotic nuclei (B). Data are from 3 replicate cultures.

**Supplementary Fig. 9: Bcl2 over-expression provides partial protection from CP-dn-ATF5 treatment.**  **A,B**. T98G cells were transfected with either GFP or GFP-BCL2 for 1 d and then exposed to 100 µM CP-dn-ATF5 for 3 additional d. The cells were then stained with Hoescht 33328 to visualize nuclei and immunostained for GFP expression and assessed for (A) relative numbers of surviving GFP+ cells (in each case normalized to its respective control) or (B) % of GFP+ cells with apoptotic nuclei. Data are from 3 independent experiments, each carried out in triplicate.
